# GasPhos: Protein Phosphorylation Site Prediction Using a New Feature Selection Approach with a GA-Aided Ant Colony System

**DOI:** 10.3390/ijms21217891

**Published:** 2020-10-24

**Authors:** Chi-Wei Chen, Lan-Ying Huang, Chia-Feng Liao, Kai-Po Chang, Yen-Wei Chu

**Affiliations:** 1 Department of Computer Science and Engineering, National Chung-Hsing University, Taichung City 402, Taiwan; d103056006@mail.nchu.edu.tw; 2 Institute of Genomics and Bioinformatics, National Chung Hsing University, Taichung City 402, Taiwan; g108019004@mail.nchu.edu.tw (L.-Y.H.); g100019010@mail.nchu.edu.tw (C.-F.L.); 3 Ph.D. Program in Medical Biotechnology, National Chung Hsing University, Taichung City 402, Taiwan; 4 Department of Pathology, China Medical University Hospital, Taichung 404, Taiwan; 5 Institute of Molecular Biology, National Chung Hsing University, Taichung City 402, Taiwan; 6 Agricultural Biotechnology Center, National Chung Hsing University, Taichung City 402, Taiwan; 7 Biotechnology Center, National Chung Hsing University, Taichung City 402, Taiwan; 8 Program in Translational Medicine, National Chung Hsing University, Taichung City 402, Taiwan; 9 Rong Hsing Research Center for Translational Medicine, National Chung Hsing University, Taichung City 402, Taiwan

**Keywords:** phosphorylation, kinase, ant colony system, genetic algorithms, feature selection

## Abstract

Protein phosphorylation is one of the most important post-translational modifications, and many biological processes are related to phosphorylation, such as DNA repair, transcriptional regulation and signal transduction and, therefore, abnormal regulation of phosphorylation usually causes diseases. If we can accurately predict human phosphorylation sites, this could help to solve human diseases. Therefore, we developed a kinase-specific phosphorylation prediction system, GasPhos, and proposed a new feature selection approach, called Gas, based on the ant colony system and a genetic algorithm and used performance evaluation strategies focused on different kinases to choose the best learning model. Gas uses the mean decrease Gini index (MDGI) as a heuristic value for path selection and adopts binary transformation strategies and new state transition rules. GasPhos can predict phosphorylation sites for six kinases and showed better performance than other phosphorylation prediction tools. The disease-related phosphorylated proteins that were predicted with GasPhos are also discussed. Finally, Gas can be applied to other issues that require feature selection, which could help to improve prediction performance.

## 1. Introduction

Protein phosphorylation is an important post-translational modification in eukaryotes [1]. It plays a key role in many biological processes, including DNA repair, transcriptional regulation, apoptosis, immune responses, signal transmission, metabolism and cell differentiation [2]. Phosphorylation is catalyzed by a specific kinase that transfers a phosphate group to the target protein and mainly occurs on serine (S), threonine (T) and tyrosine (Y) residues. Approximately one-third to one-half of proteins can be phosphorylated in eukaryotic cells [3]. Furthermore, Manning et al. [4] confirmed that there are 518 protein kinases in the human body, of which 244 protein kinases are related to cancer and other diseases. Therefore, accurately identifying the substrates of phosphorylation and phosphorylation sites will help reveal the molecular mechanism of phosphorylation-related biological processes and solve related diseases [5,6].

Although mass spectrometry can provide a large amount of phosphorylation data, this technique cannot identify kinase-specific protein phosphorylation sites and requires very expensive equipment and expertise. Therefore, many phosphorylation prediction tools have been proposed to predict specific kinases; these tools utilize machine learning predictions [7,8,9,10], such as Muste [11], KinasePhos 2.0 [12] and PPSP [13]. Among them, Muste uses nearest neighbor method scores, disorder scores and amino acid frequencies as features, while KinasePhos 2.0 uses sequences and coupling patterns to identify phosphorylation sites; both technologies are trained by a support vector machine. PPSP uses Bayesian decision theory to predict phosphorylation sites. In addition, biological properties are also used for prediction, such as with the GPS [14] and iGPS [15]; GPS uses the motif length selection (MLS) method, and iGPS is based on GPS [16] and adds information such as protein interactions to predict phosphorylation sites. Other prediction tools are non-specific [17], such as NetPhos [18] and DISPHOS [19]. NetPhos uses neural networks to predict the phosphorylation sites, while DISPHOS uses the amino acid frequency and disorder information to identify phosphorylation sites.

To improve the performance of human phosphorylation site prediction, this study integrated four protein phosphorylation databases to collect human phosphorylation sites. The kinase family and subfamily data sets were organized and evaluated for suitable classification methods from 35 machine learning methods for different kinases. The features suitable for machine learning are not the same for different kinases. Therefore, the feature selection algorithm Gas proposed in this research was used to select important features to construct the prediction model. Gas is based on the ant colony system (ACS) [20] and genetic algorithm (GA) and uses the following strategies: (i) the binary transformation strategy and state transition rule, reducing the number of overall paths, preventing pheromones from being overly dispersed in different paths, resulting in waste of pheromones and, at the same time, reducing the calculation time and proposing a new state transition rule. (ii) The mean decrease Gini index (MDGI) is used as a heuristic value [21,22,23] to help ants obtain better solutions faster. (iii) The pheromone update rule was used with a local update to escape the best solution for the local optima and a global update to allow ants to select a low number of high-performance feature subsets. (iv) Genetic algorithm search assistance was used, providing better path information for ants. Through the above strategies, a new feature selection algorithm was developed and applied to the prediction of protein phosphorylation sites.

In the analysis and evaluation of the system, we considered the number of data sets and chose CDK (S/T), CK2 (S/T), MAPK (S/T), PKA (S/T), PKC (S/T) and Src (Y) for a total of six kinds of kinases to construct 11 prediction models. Compared with five kinase-specific prediction tools, GasPhos had the best prediction performance. In five-fold cross-validation, the overall average Matthew correlation coefficient reached 0.739, which was higher than those of the other five prediction tools. By analyzing the performance of each prediction tool for different functional proteins, in addition to the defense proteins, our GasPhos was better than the other five prediction tools for enzymes, contractile proteins, regulatory proteins and receptor proteins. In addition, the features selected by Gas were related to amino acid frequency. Moreover, analyzing important features and their physical and chemical properties may also be the key to phosphorylation prediction. Finally, the phosphorylation sites of human disease-related proteins and viruses were used in a case study to successfully predict these sites.

## 2. Results

### 2.1. Selection of Machine Learning Methods and Heuristic Functions

We constructed prediction models for six kinases: CDK, CK2, MAPK, PKA, PKC and Src. Tables S2–S12 shows the performance of 35 machine learning methods using all feature construction models on the six kinases, including Sn, Sp, Acc and MCC. Table 1 summarizes the best classifiers for the six kinases.

Based on the best classifier, this study compares the effectiveness of four different heuristic functions for ant colony system feature selection (ACSFS), including the mean decrease Gini index (MDGI), Pearson correlation coefficient (PCC) [24], information gain (IG) [25] and F-score [26], as shown in Table 1. It can be found that using different heuristic functions is helpful for the ant colony system. The performance of the model based on ant colony system feature selection was better than that before feature selection. Specifically, the PCC and MDGI proposed by this research had the best MCC, which reached an average of 0.700 and 0.713, respectively, especially the MDGI average MCC, which increased from 0.648 to 0.713. Therefore, we used the MDGI as the heuristic function for the ant colony system.

### 2.2. Analysis of GA-Aided Strategy

To further improve the performance of the ant colony system, we used genetic algorithms to aid the ant colony system (ACSGAFS). Table 2 shows the performance before and after adding the genetic algorithm. The ACSGAFS MCC after application of the GA was higher than the ACSFS without the GA, especially for PKA_T and Src_Y, where there was a significant increase of approximately 0.034 and 0.048 in the MCC, and the overall average performance also increased, as indicated by an MCC of 0.013. The results showed that the application of the GA can indeed improve the performance of the ant colony system and, finally, we named this method Gas.

### 2.3. Full Pseudo-Random Proportional Rule

From Table 3, we can see that the MCC values of the pseudo-random proportional rule indicated a lower performance than that of the binary transformation strategy. Therefore, we proposed a full pseudo-random proportional rule to improve this problem. The average performance of our full pseudo-random proportional rules reached an MCC value 0.739, which is better than the 0.720 of the pseudo-random proportional rule. The full pseudo-random proportional rule can effectively reduce the number of features. The average uses 38.64 features, which is nearly half of the 76.64 features used in the pseudo-random proportional rule. Therefore, in the end, we use the full pseudo-random proportional rule in our proposed method.

### 2.4. Comparison with Other Feature Selection Methods

We compared Gas with the genetic algorithm and a simulated annealing algorithm. Table 4 lists the MCC values of Gas and the other two feature selection methods. Separately, we used a simulated annealing algorithm and genetic algorithm, tested the same training set and classification algorithm as used in Gas, and obtained an average MCC of 0.649 and 0.696 in the five-fold cross-validation. Using the feature subset selected by Gas, the classification performance was better than that of the other two methods. The overall average MCC was 0.739. Gas uses heuristic functions to enable ants to explore better answers at the beginning, which is conducive to subsequent exploration and better results. Afterwards, the genetic algorithm evolves based on the ant’s answer, and the result of the evolution is fed back to the ant. Combining the characteristics of the two algorithms can achieve better results than using a single algorithm.

### 2.5. Comparison with the Existing Predictors

The Gas method we proposed was used to construct a phosphorylation site prediction model named GasPhos. Finally, the performance of GasPhos was compared with that of other phosphorylation site prediction tools, including KinasPhos 2.0, GPS, iGPS, Muste and PPSP. As shown in Table 5, our proposed GasPhos method had a performance represented by an average MCC of 0.739, which was better than that of other tools. The MCC of GasPhos was 0.463 points higher than that of KinasePhos2.0, which is a eukaryotic phosphorylation prediction tool. Among GPS, iGPS, Muste and PPSP, which are human phosphorylation prediction tools, the highest MCC was 0.621 for PPSP, while that of GasPhos was 0.118 points higher.

## 3. Discussion

### 3.1. Similarity of Conserved Sequences and Features

**Figure 1:**
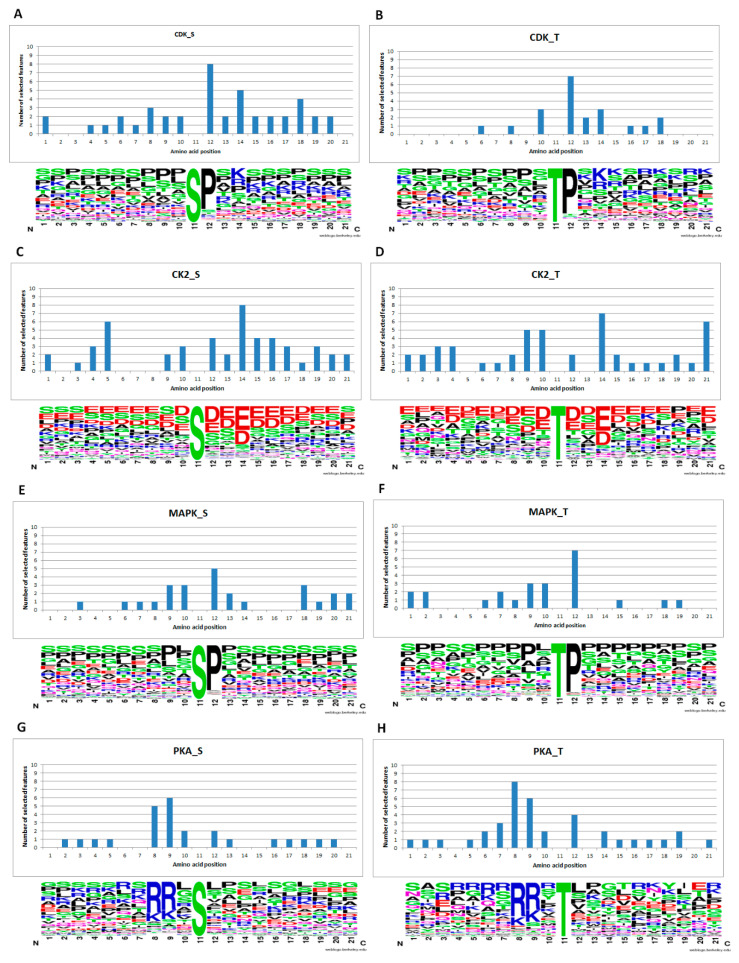
Comparison of conserved sequences and feature subsets.

We used WebLogo [27] to calculate the frequency of the amino acids of six kinases in 11 training sets with P4H in a specific position. The higher the frequency, the larger the letter size, and vice versa. The amino acid frequency and feature selection results obtained with Gas are shown in Figure 1 and Figure S1. For CDK_S, CDK_T, MAPK_S and MAPK_T, there is usually a proline in which the amino acid next to the phosphorylation site on the right corresponds to the Gas feature selection results, also showing that the position is important. Similarly, the third amino acid downstream of the phosphorylation site of CK2_S and CK2_T was frequently aspartic acid and glutamic acid. Therefore, the number of features selected for this location was greater than that for other locations. In the case of PKA_S and PKA_T, the second and third amino acids upstream of the phosphorylation site also showed the same situation. These results show that the Gas feature selection proposed in this study can select important feature subsets to help the classification algorithm improve its prediction efficiency.

In the feature results selected by Gas, there were four major biological features: (i) hydrophobicity. Huang et al. [28] proposed that phosphorylation often occurs in low-hydrophobicity regions, so there is a higher possibility of phosphorylation of residues in low-hydrophobicity regions. (ii) Electrostatic charge. Because the phosphate group has a strong negative charge, these groups will repel each other when there are many negatively charged residues. As a result, there are fewer negative amino acids around the phosphorylation site. (iii) Side chain length. The side chain structure of amino acids will affect their hydrophobicity and charge. Therefore, we suppose that side chain lengths may have a correlation with (i) and (ii). (vi) Number of codons. The number of codons affects the stability of heredity. The greater the number of codons is, the lower the probability of encoded amino acid changes when a mutation occurs. Thus, when a region mainly comprises amino acids which have a larger number of codons, the sequence retention is also higher, and the probability of phosphorylation may also be greater. As a result, this feature may help reveal the connection between positions near the phosphorylation site. Therefore, the features selected by Gas are related to phosphorylation and can improve the accuracy of prediction. Even so, these features still need to be based on more studies, and more verification is needed to support them.

### 3.2. Performance for Different Functional Proteins

To assess the predictive performance of the tool for proteins with different functions, we used the 1148p testing data to divide the proteins into defense proteins, enzymes, contractile proteins, regulatory proteins, receptor proteins and other functional proteins. Table 6 shows the MCC values of GasPhos and other tools for different functional proteins. In terms of the effectiveness of defense proteins, GPS can obtain an MCC of 0.499; thus, GPS is more suitable for the prediction of defense protein phosphorylation than the other models. The MCC of GasPhos for defense proteins was 0.396 because the proportion of defense proteins was lowest in our five types of functional protein training data. If we can increase the defense protein data, this may improve the accuracy of the prediction. For the other five functional classifications, GasPhos was more efficient than the other tools. As a result, GasPhos is suitable for the prediction of proteins with these functions

### 3.3. Case Study Predicting the Phosphorylation Sites of Disease-Related Proteins

In this study, human RAD9 protein, histone deacetylase 1 (HDAC1), HIV-1 viral protein U (Vpu) and IVA nucleoprotein (NP) were used as case studies. Rad9 is involved in many important biological functions, including DNA repair and induction of apoptosis. Apoptosis is regulated by phosphorylation of RAD9 by cyclin A-Cdk2 [29]. In addition, a more recent study has shown [30,31] that HDAC1 may play an important role in tumor formation, phosphorylating a specific location of HDAC1 by CK2 to regulate its activity and cause tumors. Vpu is a small membrane phosphoprotein that can be phosphorylated by CK-2 at S52 and S56, resulting in recruitment of beta-transducin repeat-containing proteins (β-TrCPs). It functions as a liaison between its target protein and ubiquitin ligase machinery, which leads to protein degradation. NP regulates the expression of different life cycles through different phosphorylation sites, while phosphorylation and dephosphorylation of Y78 regulate replication, transcription and NP nuclear export. RAD9 is mentioned in the literature [32], with the five sites of S277, S328, S336, T292 and T355 being phosphorylated, and the CDK_S and CDK_T models successfully predicted these five sites. In the case of HDAC1 and Vpu, three phosphorylated sites on HDAC1 (S393, S421 and S423) and two phosphorylated sites on Vpu (S52, S56) were identified in the literature [33]. In the same way, the CK2_S model has also successfully predicted these points. NP was found to be phosphorylated by Src, and it was also successfully predicted by the Src_Y model. These results prove that our proposed GasPhos method is reliable.

## 4. Materials and Methods 

### 4.1. Data Preparation

The experimental data of this study were extracted from four protein phosphorylation databases, UniProtKB/Swiss-Prot [34], Phospho.ELM [35], PhosphoSitePlus [36] and PhosphoPOINT [37]. We extracted experimentally verified human phosphorylation sites, protein sequences and kinase information from these databases and collected these data to create a new data set named P4H. We referred to the kinase classification information provided by Lee et al. [38] and classified P4H according to kinase family or subfamily, and the amount of data was greater for CDK (S/T), CK2 (S/T), MAPK (S/T), PKA (S/T), PKC (S/T) and Src (Y). The modified residues S, T and Y were divided into 11 data sets, and 11 kinase prediction models were constructed. CD-HIT [39] was used to remove redundant and similar sequences to avoid overestimation with the prediction model. The threshold was set to 0.7. The detailed numbers are listed in Table S1. In addition, to encode amino acid information around phosphorylated residues, we used a window size of 21 excised sequence fragments [40]. If the S, T or Y residues on the protein sequence were annotated as phosphorylated sites in the database, the sequence fragments consisting of them and the left and right 10 amino acids were included as positive data. The rest of the sequence fragments that were not annotated as phosphorylation sites were used as negative data. However, the amount of negative data was much larger than that of positive data, and even in the CDK_S area, the gap was close to 40-fold. If all the negative data were added to the training set for use, it may have led to biased prediction results and make the prediction results inaccurate. Therefore, in our research, we used random methods to obtain the same amount of negative data as positive data and created a training data set with a 1:1 ratio. In addition, a data set called 1148p was created according to the types of proteins in the 11-kinase training set, which was used to evaluate the performance of different tools for different functional proteins, including defense proteins, enzymes, contractile proteins, regulatory proteins, receptor proteins and other functional proteins.

### 4.2. Feature Encoding

In previous studies, most of the codes for the physical and chemical properties of amino acids used the Amino Acid Index Database (AAindex) [41], which contains 544 biochemical and physical properties of amino acids. However, the use of 544 features for encoding would be excessive and make the calculation too slow. Therefore, only 10 integrated physical and chemical properties were used in this study. These were in accordance with those of William et al. [42], who used similarity to simplify the range of amino acid characteristics, integrating the AAindex features into polarity, secondary structure, molecular size or volume, and five codon characteristics of diversity and electrostatic charge. In addition, Mathura et al., 2001 [43] sorted out and summarized five characteristics from the literature: hydrophobicity, side chain length, α-helix propensity, number of codons and β-strand propensity. In this study, the above 10 characteristics were used for encoding, and each of these characteristics had values corresponding to 20 amino acids. We normalized these values from 0.0 to 1.0 and then used them for coding. An amino acid was represented by 10 values. Finally, the number of features was the number of amino acids in the sequence fragment multiplied by a vector of 10 dimensions, and there were 200 features in total.

### 4.3. Evaluating Machine Learning Methods

Different problems and data are suitable for different machine learning methods. To select suitable classification algorithms for the 11 training sets, we used 35 classification methods from six broad categories, including tree, rule, meta, lazy, function and Bayes methods, for performance evaluation and implemented these methods with Weka [44]. In this study, each training set was used to evaluate the prediction method with all the features and five-fold cross-validation. Finally, 11 training sets were selected to suit the classification method. To obtain better classification results, we choose the machine learning method with the highest MCC for subsequent development of Gas and designed and tested the feature selection algorithm.

### 4.4. GA-Aided Ant Colony System

**Figure 2:**
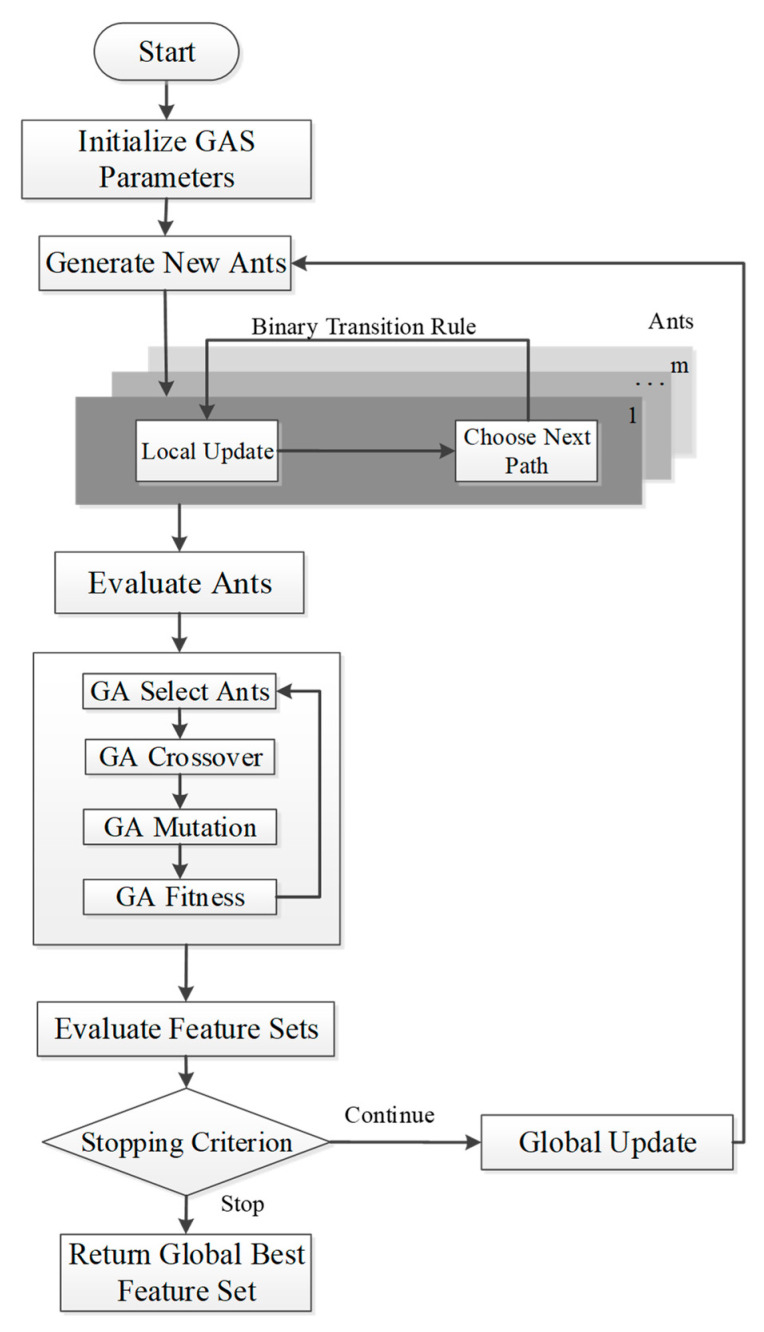
Gas flowchart.

This study proposes a new feature selection algorithm, Gas, which is based on the ant colony system and has improved path selections, heuristic values and pheromone updates. Finally, the genetic algorithm was used to aid the ants. The Gas execution process is shown in Figure 2 and Algorithm S1, and the detailed steps were as follows:

1. Initialization of Gas parameters at the beginning of the algorithm. 2. Generation of new ants and construction of candidate solutions based on the binary transformation strategy and state transition rule. After the ant selected a path, it updated the pheromone locally. 3. When all the ants completed their tour, the machine learning classifier was used to evaluate the performance of the feature subset selected by each ant, and the MCC was used to determine the ant’s ability. 4. The step 3 ant colony underwent the evolutionary process of selection, crossover and mutation through the GA until the set number of generations stopped. 5. The optimal ant was chosen as the global best ant. 6. If the set number of generations was reached, the algorithm was stopped and the feature subset selected by the global best ant was output. 7. If the set number of generations was not reached, the global best ant was used to update the global pheromone, and the process was repeated starting at step 2.

### 4.5. New Ant Colony System

The ant colony system in Gas used a new binary transformation strategy and state transition rule, which used the mean decrease Gini index (MDGI) as a heuristic value. The update of pheromones was determined by the number of feature subsets and the performance of the prediction model.

#### 4.5.1. Binary Transformation Strategy

**Figure 3:**
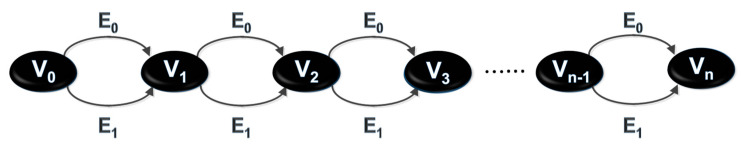
The path selection strategy adopted by Gas.

First, the path selection was different from that of the traditional ant colony system, which uses a complete graph in the application of the feature selection problem. We used the digraph to represent the application, as shown in Figure 3. Each node has only two connected paths, representing the selection ($E_{1}$) or not ($E_{0}$) of the connected nodes (features), and the ant will only choose one of the paths to move forward. When an ant completes the entire tour, the selected feature subset is determined according to the path chosen by the ant. Compared with the original method of using a complete graph, this binary transformation strategy can save considerable computational time by completing the entire tour before calculating the performance evaluation. We used a binary method to indicate whether a feature was selected. Each ant had a binary vector to represent the state of each feature. When the feature was selected, the corresponding code was 1; otherwise, it was 0. The probability that a path will be selected by an ant was calculated as follows:

(1) $$p_{i}^{j}\left(t\right)=\;\frac{{\left[\tau_{i}^{j}\left(t\right)\right]}^{\alpha }{\left(\eta_{i}^{j}\right)}^{\beta }}{{\left[\tau_{i}^{0}\left(t\right)\right]}^{\alpha }{\left(\eta_{i}^{0}\right)}^{\beta }+\;{\left[\tau_{i}^{1}\left(t\right)\right]}^{\alpha }{\left(\eta_{i}^{1}\right)}^{\beta }}\;(i=1,2,\ldots ,n;j=0,1)$$

where $\tau^{1}$ represents the pheromones of path $E_{1}$, $\tau^{0}$ is the pheromones of path of${\;E}_{0},\;\eta^{1}$ is the importance of the feature and $\eta^{0}$ is the average importance of all features. α and β represent the weights of the pheromone and heuristic values, respectively. *i* is the *i*-th feature, and *j* is the path of $E_{1}$ or $E_{0}$. Therefore, when choosing a path, the higher probability is not necessarily chosen, and low probability paths also have the opportunity to be selected.

#### 4.5.2. State Transition Rule

Furthermore, we changed the pseudo-random proportional rule of the ant colony system and proposed a new pseudo-random proportional rule called the full pseudo-random proportional rule. The original ant colony system has only two possibilities: selecting features or not selecting features to construct feature subsets. However, the full pseudo-random proportional rule we proposed adds the possibility of not selecting features to help reduce redundancy features, as follows:

(2) $$J=\{\begin{array}{c} p_{i}^{j}\left(t\right)\;if\;q>\;q_{0},\\ 0\;otherwise, \end{array}$$

When an ant moves from node V*i* to node V*j*, the probability of choosing a certain path depends on a random variable q and parameter $q_{0}\;$, both of which are between 0 and 1. Each time the ant chooses a path, a variable q is randomly generated. If q ≦$\;q_{0}$, E0 is directly selected; otherwise, if q >$\;q_{0}$, Equation (1) is used to calculate the probability of a path being selected. By setting a variable $q_{0}$ (while avoiding setting this variable as too large of a value, otherwise it may cause most of the ants to not select features directly), the ants have a certain chance of not selecting features. The advantage of this is that for some features that have a high correlation with classification, it does not affect performance or even reduces performance. These features will be selected because they have a higher heuristic value. However, with the full pseudo-random proportional rule, there is a certain probability of ignoring these features, thereby reducing the number of features and even improving the classification performance.

In addition to the use of pheromones, the ant colony system also adds a heuristic function so that ants have another basis for path selection, so ants prefer to choose important features instead of relying on pheromones. A suitable heuristic function can help ants find a better solution faster. In other related studies, there are different design methods using the information gain [25], F-score [26] and minimum redundancy maximum relevance [45] as heuristic values. In this research, the random forest calculation MDGI (R randomForest package) was used to repeat the calculation 10 times, and the heuristic value was averaged to help the ants choose the most important features and thus improve prediction performance.

#### 4.5.3. Update of Pheromones

Pheromone update depends on different update methods and different parameter settings, which will affect the future trends and convergence speed of the ant. In the ant colony system, there are two pheromone update methods: local and global. Local update means that when an ant walks a path, it will immediately change the pheromone concentration of the path so that the pheromone concentration of the path is reduced. Therefore, the probability of the next ant choosing the path taken by the previous ant will decrease slightly, so that the ant can increase the chance of exploring other paths, giving the system a chance to escape the local optima, and the pheromone of the unselected path remains unchanged. The local update was calculated according to Equation (3).

(3) $$\tau_{i}^{j}\left(t+1\right)=\left(1-p\right)\tau_{i}^{j}\left(t\right)+p\tau_{0}$$

where *p* is the pheromone evaporation rate and $\tau_{0}\;$is the initial pheromone. Another type of pheromone update is the global update. After the completion of each round of the construction process, only the global best ant (the selected feature subset with the best performance) is left with pheromones, and the path taken by the global best ant increases its pheromone concentration, so that later ants will more likely choose these paths. However, when the performance MCC is negative, it may reduce the pheromone, so that the ants do not choose these paths, while the other paths reduce the concentration of pheromones through the evaporation of pheromones. The global update was calculated according to Equation (4).

(4) $$\tau_{i}^{j}\left(t+1\right)=\left(1-p\right)\tau_{i}^{j}\left(t\right)+\Delta \tau_{i}^{j}\left(t\right)$$

where $\Delta \tau_{i}^{j}\left(t\right)\;$is the pheromone left by the ant. The concentration of the pheromone left varies according to the quality of the ant, which was calculated according to Equation (5).

(5) $$\Delta \tau_{i}^{j}\left(t\right)=\{\begin{array}{c} \rho (S^{best}\left(t\right))+\;\left(1-\frac{\ell (S^{best}\left(t\right))}{n}\right)\;if\;i\;\in S^{best}\left(t\right),\;j=1,\;\\ 0\;otherwise, \end{array}$$

where $S^{best}\left(t\right)$ represents the global best ant feature subset, $\rho (S^{best}\left(t\right))$ represents the MCC performance of the feature subset, $\ell (S^{best}\left(t\right))$ represents the feature number of the feature subset and n is the number of all features. According to this formula, when the classification efficiency of the feature subset selected by the global best ant is higher and the number of feature subsets selected is smaller, more pheromones remain. In this way, a model with high performance and a small number of features can be explored.

### 4.6. The GA Strategy

This study used genetic algorithms to aid the ant colony system. In addition to local updating, ants can also use the GA to escape the local optimal solution to help ants find better solutions. All the ants in each round complete their tour, and their performance is evaluated; then they are subjected to the GA as populations, and the GA’s selection, crossover and mutation processes are used to evolve better ants. However, the GA-derived generations are limited by its execution time, so the GA does not necessarily produce the optimal solution. Therefore, we compared the ants before and after the evolution of the GA with the best ants at present. If there was a better ant than the best ant in all the current rounds, we set it as the best ant in the global update.

### 4.7. System Implementation

**Figure 4:**
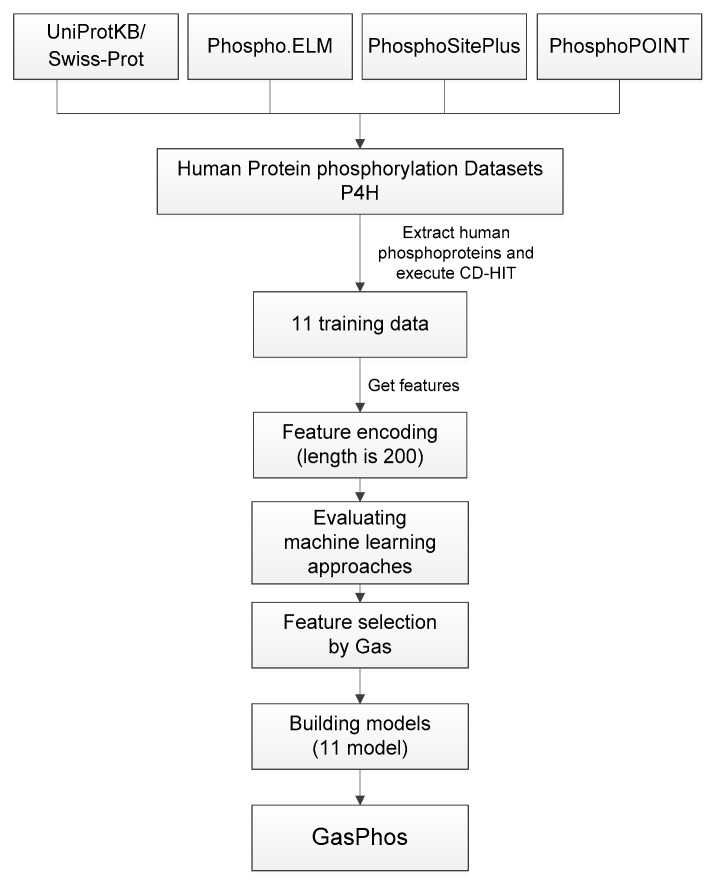
GasPhos flow chart.

The process of system implementation is shown in Figure 4. First, we collected the human phosphorylation sites, sequences and kinases that were identified in the four phosphate databases and extracted the data of six kinase families or subfamilies, which were divided into 11 categories according to their residues. After the generation of each data set, CD-HIT was used to remove duplicate and similar sequences with a threshold of 0.7, and then sequence fragments were created with a window size of 21. Then, the training set was created with a ratio of positive to negative numbers of 1:1. Then, amino acids with 10 physical and chemical properties were encoded, the fixed residues in the center of the sequence fragment were removed and the feature length after encoding was 200. Then, the machine learning algorithm provided by Weka was used to evaluate 35 methods for each training set. From these 35 types of machine learning, the method with the highest MCC for each training set was selected for later evaluation of feature selection. After confirming the optimal classification method of each data set, the Gas method proposed in this research was used to perform feature selection. The parameters used in Gas were set as follows: iterations 10, ant number 30, alpha 0.8, beta 2.0, initial pheromone 1 in the ACS, pheromone evaporation rate 0.2, q 0:0.35. In the GA part, the parameters were as follows: crossover rate 0.7, mutation rate 0.1, population 30, chromosome length 200, generations 10 and, finally, the subset of features selected by Gas was used to build the GasPhos model.

### 4.8. Evaluation of Classification Performance

To evaluate the performance of the classifier, four measurements, including sensitivity (Sn), specificity (Sp), accuracy (Acc) and Matthews correlation coefficient (MCC), were used to evaluate the classified predictive power, where Sn, Sp and Acc represented the positive, negative and overall data sets predicting success rates, respectively. MCC was used for evaluating the correlation of positive and negative data accuracy, and its value lies between −1 and 1; the closer the value to 1, the more accurate the predictions, the closer the value to −1, the more inaccurate the prediction. To avoid single positive or negative data sets having too high accuracy, leading to higher overall predictive accuracy and thus affecting credibility, the MCC provided a better evaluation. These measurements were thus calculated according to the following equations:

(6) $$\mathrm{Sn}=\frac{TP}{TP+FN}$$

(7) $$\mathrm{Sp}=\frac{TN}{TN+FP}$$

(8) $$\mathrm{Acc}=\frac{TP+TN}{TN+FN+TP+FP}$$

(9) $$\mathrm{MCC}=\frac{\left(TP\times TN\right)-\left(FP\times FN\right)}{\sqrt{\left(TP+FN\right)\times \left(TN+FP\right)\times \left(TP+FP\right)\times \left(TN+FN\right)}}$$

where TP, FP, TN and FN represent the correct prediction of positives, the incorrect prediction of negatives, the correct prediction of negatives and the incorrect prediction of positives, respectively.

## 5. Conclusions

This study proposes a prediction system, GasPhos, for predicting human protein phosphorylation sites and constructs prediction models for the six protein phosphorylation kinase families of CDK, CK2, MAPK, PKA, PKC and Src. Because the number of other kinase families or subfamilies is relatively small, we constructed prediction models for predicting non-specific phosphorylation sites. The overall architecture is mainly to evaluate the performance of the classifier for different human kinase phosphorylation data. After selecting the best machine learning method, the Gas algorithm was used for feature selection to improve prediction performance. The results showed that our strategy of fusing the two algorithms can obtain better performance than other phosphorylation prediction tools that use a single strategy. The overall average Matthews correlation coefficient reached 0.739, which was higher than those of the other tools. This shows that our system is more effective than other tools in predicting human phosphorylation sites, and the overall predictive capability has higher accuracy than other tools. We used human RAD9 protein, histone deacetylase 1 (HDAC1), HIV-1 viral protein U (Vpu) and IVA nucleoprotein (NP) as case studies to explore the applicability of this system. HDACs are very important to convert chromatin states and transition histones–protamines in human spermatozoa. GasPhos could be used to study the effects of heavy metals that lead to alterations in the reproductive health of marine organisms and humans [46,47,48].

In addition, Gas also had better performance in the measurement of the MCC than the genetic algorithm and simulated annealing algorithm. This also shows that the feature selection Gas proposed in this research and the selected feature subsets are more effective for machine learning than the use of all available features. In addition, the number of features after feature selection was reduced by approximately four-fifths, on average. Therefore, the feature selection algorithm designed in this study is helpful for machine learning methods in constructing prediction models. Finally, the algorithm proposed in this study can also be applied to other topics with feature selection requirements or other research fields and is expected to help solve various problems. In terms of phosphorylation site prediction, our research shows that the proposed prediction model has better performance in the prediction of human kinase-specific protein phosphorylation sites than other models. GasPhos can predict the phosphorylation sites of six kinds of protein kinases, integrate other kinase data and build a model to predict non-specific protein phosphorylation sites. Although there are many categories and few data, the accuracy rate can also be 60% or higher (Table S13). GasPhos is freely accessible to the public at http://predictor.nchu.edu.tw/GasPhos.

## Figures and Tables

**Table 1:** Comparison of different heuristic functions.

Kinase	Classifier	All Features	ACSFS			
			IG	F-Score	PCC	MDGI
CDK_S	BFTree	0.711	0.723	0.747	0.748	0.746
CDK_T	SimpleCart	0.764	0.770	0.767	0.773	0.776
CK2_S	NaiveBayes	0.630	0.699	0.692	0.702	0.700
CK2_T	MultiBoostAB	0.625	0.630	0.681	0.682	0.692
MAPK_S	BFTree	0.742	0.745	0.770	0.775	0.773
MAPK_T	BFTree	0.843	0.850	0.859	0.849	0.854
PKA_S	DecisionTable	0.747	0.769	0.772	0.781	0.781
PKA_T	RBFNetwork	0.720	0.763	0.800	0.823	0.862
PKC_S	DecisionTable	0.558	0.574	0.585	0.585	0.585
PKC_T	SimpleLogistic	0.470	0.502	0.579	0.579	0.649
Src_Y	NaiveBayes	0.320	0.359	0.394	0.408	0.421
Avg.		0.648	0.671	0.695	0.700	0.713

**Table 2:** The results of the genetic algorithm (GA)-aided strategy with the mean decrease Gini index (MDGI).

Kinase	ACSFS				ACSGAFS			
	SN	SP	ACC	MCC	SN	SP	ACC	MCC
CDK_S	0.838	0.906	0.872	0.746	0.845	0.904	0.874	0.751
CDK_T	0.861	0.913	0.887	0.776	0.865	0.913	0.889	0.779
CK2_S	0.814	0.884	0.849	0.700	0.815	0.898	0.856	0.716
CK2_T	0.813	0.875	0.844	0.692	0.825	0.875	0.850	0.705
MAPK_S	0.877	0.894	0.885	0.773	0.877	0.901	0.889	0.779
MAPK_T	0.904	0.949	0.927	0.854	0.914	0.944	0.929	0.859
PKA_S	0.871	0.907	0.889	0.781	0.878	0.902	0.890	0.782
PKA_T	0.905	0.951	0.929	0.862	0.888	1.000	0.944	0.896
PKC_S	0.795	0.789	0.792	0.585	0.804	0.786	0.795	0.592
PKC_T	0.815	0.831	0.823	0.649	0.792	0.862	0.827	0.656
Src_Y	0.715	0.704	0.709	0.421	0.752	0.715	0.733	0.469
Avg.	0.837	0.873	0.855	0.713	0.841	0.882	0.862	0.726

**Table 3:** The result of the full pseudo-random proportional rule.

Kinase	Binary Transformation Strategy	Pseudo-Random Proportional Rule	Full Pseudo-Random Proportional Rule
CDK_S	0.751 (50)	0.743 (104)	0.755 (33)
CDK_T	0.779 (41)	0.779 (43)	0.792 (27)
CK2_S	0.716 (74)	0.712 (112)	0.712 (49)
CK2_T	0.705 (71)	0.705 (50)	0.758 (44)
MAPK_S	0.779 (45)	0.769 (83)	0.786 (30)
MAPK_T	0.859 (29)	0.866 (27)	0.864 (20)
PKA_S	0.782 (45)	0.774 (62)	0.795 (25)
PKA_T	0.896 (55)	0.896 (40)	0.909 (34)
PKC_S	0.592 (77)	0.585 (126)	0.615 (48)
PKC_T	0.656 (73)	0.651 (64)	0.667 (54)
Src_Y	0.469 (92)	0.437 (132)	0.477 (61)
Avg.	0.726 (59.27)	0.720 (76.64)	0.739 (38.64)

The number in parentheses is the number of features selected.

**Table 4:** The results of different feature selection methods.

Kinase	Simulated Annealing Algorithm	Genetic Algorithm	Gas
CDK_S	0.692	0.736	0.755
CDK_T	0.764	0.773	0.792
CK2_S	0.602	0.699	0.712
CK2_T	0.580	0.694	0.758
MAPK_S	0.746	0.760	0.786
MAPK_T	0.844	0.844	0.864
PKA_S	0.770	0.780	0.795
PKA_T	0.769	0.830	0.909
PKC_S	0.571	0.589	0.615
PKC_T	0.379	0.557	0.667
Src_Y	0.323	0.391	0.477
Avg.	0.640	0.696	0.739

**Table 5:** The results of different predictors for various specific kinases.

Kinase	KinasPhos 2.0	GPS	iGPS	Musite	PPSP	GasPhos
CDK_S	0.150	0.593	0.503	0.677	0.689	0.755
CDK_T	0.260	0.688	0.575	0.743	0.761	0.792
CK2_S	0.647	0.619	0.423	0.661	0.583	0.712
CK2_T	0.400	0.590	0.434	0.555	0.550	0.758
MAPK_S	0.390	0.588	0.613	0.691	0.696	0.786
MAPK_T	N/A *	0.730	0.708	0.820	0.824	0.864
PKA_S	0.161	0.765	0.516	0.747	0.747	0.795
PKA_T	0.560	0.813	0.514	0.719	0.700	0.909
PKC_S	0.166	0.464	0.466	0.493	0.521	0.615
PKC_T	0.231	0.459	0.410	0.418	0.436	0.667
Src_Y	0.075	0.459	0.329	0.285	0.319	0.477
Avg.	0.276	0.615	0.499	0.619	0.621	0.739

* N/A is not available.

**Table 6:** Proteomics analysis of various functional proteins by different predictors.

Function Type	KinasPhos 2.0	GPS	iGPS	Musite	PPSP	GasPhos
Defense proteins	0.040	0.499	0.231	0.386	0.458	0.396
Enzymes	0.252	0.606	0.515	0.543	0.544	0.680
Contractile proteins	0.064	0.247	0.306	0.414	0.391	0.447
Regulatory proteins	0.212	0.393	0.273	0.426	0.469	0.539
Receptor proteins	0.176	0.571	0.447	0.500	0.543	0.588
Other	0.269	0.602	0.473	0.627	0.610	0.744
Avg.	0.169	0.486	0.374	0.483	0.502	0.566

## Supplementary Materials

Supplementary materials can be found at https://www.mdpi.com/1422-0067/21/21/7891/s1.
