# Supplementary material for: GasPhos: Protein Phosphorylation Site Prediction Using a New Feature Selection Approach with a GA-Aided Ant Colony System
Source: Int J Mol Sci. 2020 Oct 24;21(21):7891. doi: 10.3390/ijms21217891 (PMC7660635; doi:10.3390/ijms21217891)
Supplement: Supplementary file 1 [file ijms-21-07891-s001.pdf]

Supplemental Data

Table S1. The number of human phosphorylation sites in different kinase types.

Kinase type	Positive	Negative
CDK_S	562	21395
CDK_T	310	9143
CK2_S	404	10177
CK2_T	80	1726
MAPK_S	446	14183
MAPK_T	197	3823
PKA_S	410	17600
PKA_T	63	1682
PKC_S	459	14334
PKC_T	130	3452
Src_Y	375	3879

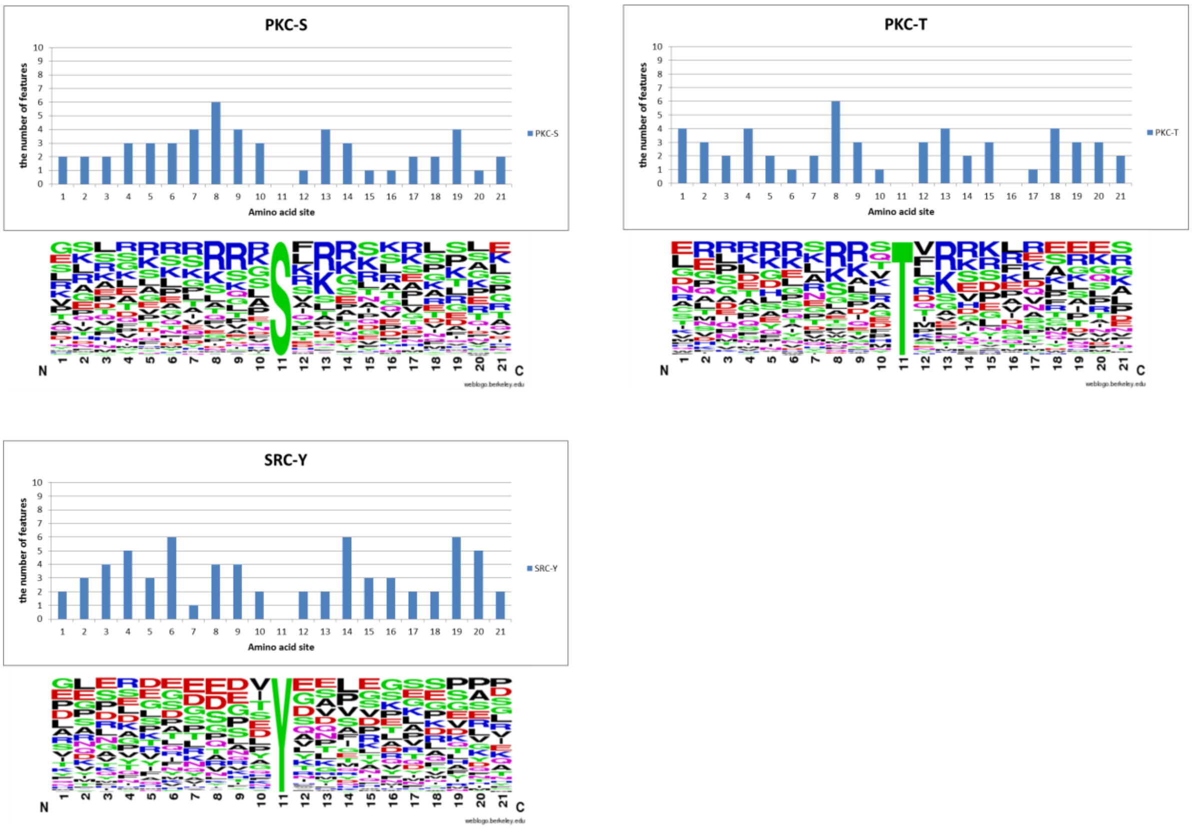

Figure S1. Comparison of conserved sequences and feature subsets.

Algorithm S1. GA-aided ant colony system.

Begin:

Initialization Gas parameters

While stopping criterion not satisfied **do**

Generate population of *m* solutions (ants)

Repeat

For each ant **do**

Choose the next node by the state transition rule

Update the pheromone locally

End for

For each individual ant $k \in m$: calculate fitness(k)

Assign *m* ant in population (chromosomes)

While $i <$ MaxGAgeneration **do**

Selection

Crossover

Mutation

Fitness calculation

End while

Determine the best global ant

Update the global pheromone

Until generations reach setting

return the feature subset selected by the global-best-ant

End while

End

Table S2. Thirty-five kinds of machine learning methods compared by CDK_S.

Classifier	Sn	Sp	Acc	MCC
SimpleCart	0.820	0.888	0.854	0.710
LibSVM	0.794	0.874	0.834	0.669
REPTree	0.824	0.843	0.834	0.667
RandomTree	0.785	0.788	0.786	0.573
RandomForest	0.843	0.858	0.851	0.701
NBTree	0.788	0.872	0.830	0.662
LMT	0.790	0.875	0.833	0.668
LADTree	0.829	0.854	0.842	0.683
J48	0.772	0.749	0.761	0.521
FT	0.797	0.779	0.788	0.577
DecisionStump	0.762	0.923	0.843	0.694
BFTree	0.813	0.895	0.854	0.711
ADTree	0.826	0.872	0.849	0.698
Ridor	0.767	0.883	0.825	0.654
PART	0.778	0.779	0.778	0.557
OneR	0.762	0.923	0.843	0.694
NNge	0.843	0.760	0.802	0.605
JRip	0.831	0.868	0.850	0.700
DecisionTable	0.785	0.897	0.841	0.686
ConjunctiveRule	0.762	0.923	0.843	0.694
AdaBoostM1	0.795	0.902	0.849	0.702
MultiBoostAB	0.762	0.923	0.843	0.694
IB1	0.728	0.587	0.657	0.318
IBK	0.728	0.587	0.657	0.318
Kstar	0.728	0.484	0.606	0.218
LWL	0.762	0.923	0.843	0.694
LibLINEAR	0.801	0.827	0.814	0.628
Logistic	0.788	0.783	0.786	0.571
MultilayerPerceptro	0.811	0.779	0.795	0.591
RBFNetwork	0.786	0.884	0.835	0.674
SimpleLogistic	0.790	0.875	0.833	0.668
SMO	0.792	0.836	0.814	0.629
SPegasos	0.452	0.899	0.675	0.392
NaiveBayes	0.810	0.881	0.845	0.692
NaiveBayesUpdateable	0.810	0.881	0.845	0.692

Table S3. Thirty-five kinds of machine learning methods compared by CDK_T.

**Classifier**	**Sn**	**Sp**	**Acc**	**MCC**
SimpleCart	0.852	0.913	0.882	0.766
LibSVM	0.858	0.852	0.855	0.710
REPTree	0.842	0.906	0.874	0.750
RandomTree	0.803	0.784	0.794	0.587
RandomForest	0.874	0.877	0.876	0.752
NBTree	0.839	0.823	0.831	0.661
LMT	0.855	0.874	0.865	0.729
LADTree	0.884	0.694	0.789	0.588
J48	0.823	0.790	0.806	0.613
FT	0.861	0.832	0.847	0.694
DecisionStump	0.842	0.919	0.881	0.764
BFTree	0.855	0.868	0.861	0.723
ADTree	0.855	0.839	0.847	0.694
Ridor	0.842	0.890	0.866	0.733
PART	0.810	0.784	0.797	0.594
OneR	0.852	0.903	0.877	0.756
NNge	0.894	0.758	0.826	0.658
JRip	0.845	0.887	0.866	0.733
DecisionTable	0.845	0.890	0.868	0.736
ConjunctiveRule	0.842	0.919	0.881	0.764
AdaBoostM1	0.877	0.861	0.869	0.739
MultiBoostAB	0.842	0.919	0.881	0.764
IB1	0.771	0.552	0.661	0.331
IBK	0.771	0.552	0.661	0.331
Kstar	0.810	0.406	0.608	0.236
LWL	0.842	0.919	0.881	0.764
LibLINEAR	0.823	0.816	0.819	0.639
Logistic	0.774	0.742	0.758	0.516
MultilayerPerceptro	0.855	0.803	0.829	0.659
RBFNetwork	0.858	0.877	0.868	0.736
SimpleLogistic	0.855	0.874	0.865	0.729
SMO	0.842	0.835	0.839	0.677
SPegasos	0.681	0.868	0.774	0.558
NaiveBayes	0.848	0.894	0.871	0.743
NaiveBayesUpdateable	0.848	0.894	0.871	0.743

Table S4. Thirty-five kinds of machine learning methods compared by CK2_S.

Classifier	Sn	Sp	Acc	MCC
SimpleCart	0.765	0.809	0.787	0.575
LibSVM	0.762	0.859	0.811	0.624
REPTree	0.730	0.817	0.774	0.549
RandomTree	0.703	0.663	0.683	0.367
RandomForest	0.832	0.740	0.786	0.574
NBTree	0.710	0.767	0.739	0.478
LMT	0.790	0.817	0.803	0.607
LADTree	0.656	0.814	0.735	0.476
J48	0.750	0.765	0.757	0.515
FT	0.755	0.757	0.756	0.512
DecisionStump	0.708	0.834	0.771	0.546
BFTree	0.748	0.804	0.776	0.553
ADTree	0.762	0.814	0.788	0.578
Ridor	0.787	0.762	0.775	0.550
PART	0.755	0.750	0.752	0.505
OneR	0.718	0.879	0.798	0.604
NNge	0.800	0.540	0.670	0.351
JRip	0.762	0.757	0.760	0.520
DecisionTable	0.767	0.817	0.792	0.585
ConjunctiveRule	0.624	0.899	0.761	0.543
AdaBoostM1	0.777	0.819	0.798	0.597
MultiBoostAB	0.705	0.832	0.769	0.541
IB1	0.752	0.686	0.719	0.439
IBK	0.752	0.686	0.719	0.439
Kstar	0.800	0.572	0.686	0.381
LWL	0.708	0.834	0.771	0.546
LibLINEAR	0.792	0.795	0.793	0.587
Logistic	0.713	0.710	0.712	0.423
MultilayerPerceptro	0.765	0.795	0.780	0.560
RBFNetwork	0.755	0.866	0.811	0.625
SimpleLogistic	0.790	0.817	0.803	0.607
SMO	0.785	0.802	0.793	0.587
SPegasos	0.480	0.906	0.693	0.427
NaiveBayes	0.760	0.866	0.813	0.630
NaiveBayesUpdateable	0.760	0.866	0.813	0.630

Table S5. Thirty-five kinds of machine learning methods compared by CK2_T.

**Classifier**	**Sn**	**Sp**	**Acc**	**MCC**
SimpleCart	0.813	0.775	0.794	0.588
LibSVM	0.650	0.825	0.738	0.482
REPTree	0.825	0.725	0.775	0.553
RandomTree	0.738	0.738	0.738	0.475
RandomForest	0.775	0.600	0.688	0.381
NBTree	0.638	0.788	0.713	0.430
LMT	0.738	0.700	0.719	0.438
LADTree	0.688	0.775	0.731	0.464
J48	0.650	0.700	0.675	0.350
FT	0.713	0.675	0.694	0.388
DecisionStump	0.813	0.775	0.794	0.588
BFTree	0.813	0.775	0.794	0.588
ADTree	0.725	0.838	0.781	0.566
Ridor	0.513	0.813	0.663	0.341
PART	0.638	0.738	0.688	0.377
OneR	0.788	0.775	0.781	0.563
NNge	0.750	0.500	0.625	0.258
JRip	0.763	0.700	0.731	0.463
DecisionTable	0.775	0.763	0.769	0.538
ConjunctiveRule	0.788	0.713	0.750	0.501
AdaBoostM1	0.825	0.713	0.769	0.541
MultiBoostAB	0.813	0.813	0.813	0.625
IB1	0.688	0.675	0.681	0.363
IBK	0.688	0.675	0.681	0.363
Kstar	0.688	0.575	0.631	0.264
LWL	0.813	0.763	0.788	0.576
LibLINEAR	0.663	0.725	0.694	0.388
Logistic	0.600	0.663	0.631	0.263
MultilayerPerceptro	0.725	0.725	0.725	0.450
RBFNetwork	0.713	0.800	0.756	0.514
SimpleLogistic	0.738	0.675	0.706	0.413
SMO	0.650	0.738	0.694	0.389
SPegasos	0.650	0.675	0.663	0.325
NaiveBayes	0.788	0.813	0.800	0.600
NaiveBayesUpdateable	0.788	0.813	0.800	0.600

Table S6. Thirty-five kinds of machine learning methods compared by MAPK_S.

Classifier	Sn	Sp	Acc	MCC
SimpleCart	0.841	0.897	0.869	0.739
LibSVM	0.843	0.879	0.861	0.722
REPTree	0.834	0.881	0.858	0.716
RandomTree	0.751	0.756	0.753	0.507
RandomForest	0.852	0.868	0.860	0.720
NBTree	0.830	0.821	0.825	0.650
LMT	0.843	0.883	0.863	0.727
LADTree	0.874	0.857	0.865	0.731
J48	0.800	0.812	0.806	0.612
FT	0.760	0.787	0.774	0.547
DecisionStump	0.839	0.901	0.870	0.741
BFTree	0.861	0.881	0.871	0.742
ADTree	0.850	0.870	0.860	0.720
Ridor	0.807	0.888	0.848	0.697
PART	0.758	0.765	0.761	0.522
OneR	0.839	0.901	0.870	0.741
NNge	0.857	0.827	0.842	0.684
JRip	0.852	0.863	0.858	0.715
DecisionTable	0.841	0.895	0.868	0.736
ConjunctiveRule	0.839	0.901	0.870	0.741
AdaBoostM1	0.841	0.886	0.863	0.727
MultiBoostAB	0.841	0.895	0.868	0.736
IB1	0.738	0.623	0.680	0.363
IBK	0.738	0.623	0.680	0.363
Kstar	0.733	0.462	0.598	0.203
LWL	0.839	0.901	0.870	0.741
LibLINEAR	0.825	0.812	0.818	0.637
Logistic	0.729	0.753	0.741	0.482
MultilayerPerceptro	0.827	0.807	0.817	0.635
RBFNetwork	0.832	0.881	0.857	0.714
SimpleLogistic	0.843	0.883	0.863	0.727
SMO	0.832	0.848	0.840	0.679
SPegasos	0.312	0.944	0.628	0.330
NaiveBayes	0.836	0.890	0.863	0.728
NaiveBayesUpdateable	0.836	0.890	0.863	0.728

Table S7. Thirty-five kinds of machine learning methods compared by MAPK_T.

**Classifier**	**Sn**	**Sp**	**Acc**	**MCC**
SimpleCart	0.904	0.939	0.921	0.843
LibSVM	0.909	0.868	0.888	0.777
REPTree	0.904	0.929	0.916	0.833
RandomTree	0.741	0.736	0.739	0.477
RandomForest	0.873	0.898	0.886	0.772
NBTree	0.853	0.878	0.865	0.731
LMT	0.904	0.929	0.916	0.833
LADTree	0.827	0.914	0.871	0.744
J48	0.868	0.832	0.850	0.701
FT	0.863	0.832	0.848	0.696
DecisionStump	0.904	0.939	0.921	0.843
BFTree	0.904	0.939	0.921	0.843
ADTree	0.898	0.929	0.914	0.828
Ridor	0.898	0.904	0.901	0.802
PART	0.838	0.863	0.850	0.701
OneR	0.904	0.934	0.919	0.838
NNge	0.909	0.904	0.906	0.812
JRip	0.898	0.934	0.916	0.833
DecisionTable	0.898	0.934	0.916	0.833
ConjunctiveRule	0.904	0.934	0.919	0.838
AdaBoostM1	0.904	0.868	0.886	0.772
MultiBoostAB	0.904	0.939	0.921	0.843
IB1	0.807	0.624	0.716	0.439
IBK	0.807	0.624	0.716	0.439
Kstar	0.822	0.462	0.642	0.305
LWL	0.904	0.939	0.921	0.843
LibLINEAR	0.853	0.858	0.855	0.711
Logistic	0.787	0.761	0.774	0.548
MultilayerPerceptro	0.817	0.807	0.812	0.624
RBFNetwork	0.888	0.929	0.909	0.818
SimpleLogistic	0.904	0.929	0.916	0.833
SMO	0.843	0.853	0.848	0.695
SPegasos	0.751	0.802	0.777	0.554
NaiveBayes	0.893	0.944	0.919	0.839
NaiveBayesUpdateable	0.893	0.944	0.919	0.839

Table S8. Thirty-five kinds of machine learning methods compared by PKA_S.

Classifier	Sn	Sp	Acc	MCC
SimpleCart	0.893	0.851	0.872	0.745
LibSVM	0.820	0.902	0.861	0.724
REPTree	0.893	0.837	0.865	0.730
RandomTree	0.793	0.815	0.804	0.607
RandomForest	0.868	0.851	0.860	0.720
NBTree	0.807	0.798	0.802	0.605
LMT	0.849	0.863	0.856	0.712
LADTree	0.846	0.820	0.833	0.666
J48	0.834	0.822	0.828	0.656
FT	0.798	0.863	0.830	0.662
DecisionStump	0.705	0.888	0.796	0.603
BFTree	0.893	0.851	0.872	0.745
ADTree	0.868	0.832	0.850	0.700
Ridor	0.834	0.880	0.857	0.715
PART	0.824	0.834	0.829	0.659
OneR	0.712	0.878	0.795	0.599
NNge	0.871	0.668	0.770	0.550
JRip	0.820	0.883	0.851	0.704
DecisionTable	0.861	0.885	0.873	0.747
ConjunctiveRule	0.673	0.920	0.796	0.612
AdaBoostM1	0.844	0.878	0.861	0.722
MultiBoostAB	0.854	0.841	0.848	0.695
IB1	0.780	0.661	0.721	0.445
IBK	0.780	0.661	0.721	0.445
Kstar	0.834	0.483	0.659	0.339
LWL	0.737	0.871	0.804	0.613
LibLINEAR	0.834	0.841	0.838	0.676
Logistic	0.763	0.785	0.774	0.549
MultilayerPerceptro	0.839	0.815	0.827	0.654
RBFNetwork	0.795	0.893	0.844	0.691
SimpleLogistic	0.849	0.863	0.856	0.712
SMO	0.829	0.829	0.829	0.659
SPegasos	0.654	0.890	0.772	0.560
NaiveBayes	0.776	0.907	0.841	0.689
NaiveBayesUpdateable	0.776	0.907	0.841	0.689

Table S9. Thirty-five kinds of machine learning methods compared by PKA_T.

Classifier	Sn	Sp	Acc	MCC
SimpleCart	0.825	0.873	0.849	0.699
LibSVM	0.810	0.810	0.810	0.619
REPTree	0.778	0.889	0.833	0.671
RandomTree	0.730	0.746	0.738	0.476
RandomForest	0.873	0.730	0.802	0.609
NBTree	0.746	0.635	0.690	0.383
LMT	0.762	0.841	0.802	0.605
LADTree	0.825	0.841	0.833	0.667
J48	0.794	0.841	0.817	0.636
FT	0.778	0.810	0.794	0.588
DecisionStump	0.794	0.921	0.857	0.720
BFTree	0.841	0.873	0.857	0.715
ADTree	0.810	0.778	0.794	0.588
Ridor	0.794	0.873	0.833	0.669
PART	0.746	0.825	0.786	0.573
OneR	0.794	0.889	0.841	0.686
NNge	0.841	0.683	0.762	0.531
JRip	0.841	0.762	0.802	0.605
DecisionTable	0.810	0.873	0.841	0.684
ConjunctiveRule	0.714	0.905	0.810	0.631
AdaBoostM1	0.810	0.762	0.786	0.572
MultiBoostAB	0.794	0.873	0.833	0.669
IB1	0.778	0.635	0.706	0.417
IBK	0.778	0.635	0.706	0.417
Kstar	0.857	0.413	0.635	0.301
LWL	0.794	0.905	0.849	0.703
LibLINEAR	0.778	0.762	0.770	0.540
Logistic	0.730	0.730	0.730	0.460
MultilayerPerceptro	0.794	0.730	0.762	0.525
RBFNetwork	0.810	0.905	0.857	0.718
SimpleLogistic	0.762	0.810	0.786	0.572
SMO	0.762	0.730	0.746	0.492
SPegasos	0.762	0.714	0.738	0.477
NaiveBayes	0.778	0.873	0.825	0.654
NaiveBayesUpdateable	0.778	0.873	0.825	0.654

Table S10. Thirty-five kinds of machine learning methods compared by PKC_S.

Classifier	Sn	Sp	Acc	MCC
SimpleCart	0.739	0.778	0.758	0.517
LibSVM	0.684	0.830	0.757	0.520
REPTree	0.721	0.767	0.744	0.489
RandomTree	0.680	0.643	0.661	0.323
RandomForest	0.795	0.686	0.741	0.484
NBTree	0.686	0.715	0.700	0.401
LMT	0.741	0.763	0.752	0.503
LADTree	0.678	0.819	0.748	0.502
J48	0.721	0.675	0.698	0.397
FT	0.706	0.736	0.721	0.442
DecisionStump	0.464	0.895	0.680	0.398
BFTree	0.702	0.810	0.756	0.515
ADTree	0.723	0.765	0.744	0.488
Ridor	0.758	0.667	0.712	0.427
PART	0.712	0.669	0.691	0.382
OneR	0.481	0.871	0.676	0.383
NNge	0.893	0.192	0.542	0.119
JRip	0.747	0.754	0.751	0.501
DecisionTable	0.773	0.784	0.779	0.558
ConjunctiveRule	0.407	0.911	0.659	0.368
AdaBoostM1	0.719	0.795	0.757	0.516
MultiBoostAB	0.525	0.856	0.691	0.404
IB1	0.688	0.630	0.659	0.319
IBK	0.688	0.630	0.659	0.319
Kstar	0.780	0.486	0.633	0.278
LWL	0.464	0.895	0.680	0.398
LibLINEAR	0.754	0.776	0.765	0.530
Logistic	0.712	0.699	0.706	0.412
MultilayerPerceptro	0.767	0.730	0.748	0.497
RBFNetwork	0.710	0.728	0.719	0.438
SimpleLogistic	0.747	0.763	0.755	0.510
SMO	0.745	0.767	0.756	0.512
SPegasos	0.362	0.924	0.643	0.345
NaiveBayes	0.723	0.804	0.764	0.529
NaiveBayesUpdateable	0.723	0.804	0.764	0.529

Table S11. Thirty-five kinds of machine learning methods compared by PKC_T.

Classifier	Sn	Sp	Acc	MCC
SimpleCart	0.631	0.792	0.712	0.429
LibSVM	0.631	0.815	0.723	0.454
REPTree	0.546	0.769	0.658	0.324
RandomTree	0.569	0.608	0.588	0.177
RandomForest	0.762	0.592	0.677	0.359
NBTree	0.608	0.500	0.554	0.108
LMT	0.677	0.762	0.719	0.440
LADTree	0.646	0.615	0.631	0.262
J48	0.654	0.623	0.638	0.277
FT	0.669	0.723	0.696	0.393
DecisionStump	0.423	0.862	0.642	0.317
BFTree	0.600	0.792	0.696	0.400
ADTree	0.646	0.723	0.685	0.370
Ridor	0.654	0.623	0.638	0.277
PART	0.708	0.669	0.688	0.377
OneR	0.615	0.631	0.623	0.246
NNge	0.977	0.069	0.523	0.110
JRip	0.631	0.685	0.658	0.316
DecisionTable	0.685	0.700	0.692	0.385
ConjunctiveRule	0.392	0.900	0.646	0.339
AdaBoostM1	0.692	0.738	0.715	0.431
MultiBoostAB	0.531	0.838	0.685	0.388
IB1	0.608	0.662	0.635	0.270
IBK	0.608	0.662	0.635	0.270
Kstar	0.669	0.600	0.635	0.270
LWL	0.454	0.869	0.662	0.355
LibLINEAR	0.608	0.669	0.638	0.277
Logistic	0.577	0.569	0.573	0.146
MultilayerPerceptro	0.669	0.685	0.677	0.354
RBFNetwork	0.738	0.677	0.708	0.416
SimpleLogistic	0.708	0.762	0.735	0.470
SMO	0.631	0.692	0.662	0.324
SPegasos	0.638	0.654	0.646	0.292
NaiveBayes	0.723	0.708	0.715	0.431
NaiveBayesUpdateable	0.723	0.708	0.715	0.431

Table S12. Thirty-five kinds of machine learning methods compared by Src_Y.

Classifier	Sn	Sp	Acc	MCC
SimpleCart	0.733	0.504	0.619	0.244
LibSVM	0.661	0.600	0.631	0.262
REPTree	0.603	0.496	0.549	0.099
RandomTree	0.541	0.549	0.545	0.091
RandomForest	0.709	0.493	0.601	0.208
NBTree	0.584	0.573	0.579	0.157
LMT	0.669	0.549	0.609	0.220
LADTree	0.480	0.627	0.553	0.108
J48	0.603	0.557	0.580	0.160
FT	0.589	0.536	0.563	0.126
DecisionStump	0.787	0.461	0.624	0.262
BFTree	0.731	0.475	0.603	0.212
ADTree	0.584	0.584	0.584	0.168
Ridor	0.592	0.581	0.587	0.173
PART	0.589	0.563	0.576	0.152
OneR	0.651	0.589	0.620	0.240
NNge	0.899	0.192	0.545	0.128
DTNB	0.669	0.557	0.613	0.228
DecisionTable	0.643	0.565	0.604	0.209
ConjunctiveRule	0.877	0.261	0.569	0.176
AdaBoostM1	0.605	0.584	0.595	0.189
MultiBoostAB	0.789	0.459	0.624	0.263
IB1	0.613	0.579	0.596	0.192
IBK	0.613	0.579	0.596	0.192
Kstar	0.659	0.557	0.608	0.217
LWL	0.811	0.411	0.611	0.241
LibLINEAR	0.597	0.589	0.593	0.187
Logistic	0.579	0.584	0.581	0.163
MultilayerPerceptro	0.659	0.597	0.628	0.256
RBFNetwork	0.605	0.653	0.629	0.259
SimpleLogistic	0.669	0.549	0.609	0.220
SMO	0.600	0.611	0.605	0.211
SPegasos	0.147	0.936	0.541	0.135
NaiveBayes	0.643	0.677	0.660	0.320
NaiveBayesUpdateable	0.643	0.677	0.660	0.320

Table S13. The performance of other kinases.

Kinase	Classifier	Sn	Sp	Acc	MCC
Abl_Y	OneR	0.679	0.764	0.721	0.448
ATM_S	AdaBoostM1	0.941	0.937	0.939	0.878
ATM_T	NaiveBayes	0.900	0.967	0.933	0.879
Aur_S	ConjunctiveRule	0.647	0.932	0.790	0.608
CAMK2_S	DecisionTable	0.855	0.840	0.847	0.703
CK1_S	NaiveBayes	0.775	0.817	0.796	0.594
EGFR_Y	IBk	0.860	0.824	0.842	0.692
IKK_S	IBk	0.780	0.800	0.790	0.585
InsR_Y	DecisionTable	0.184	0.960	0.572	0.200
Lck_Y	NaiveBayes	0.812	0.876	0.844	0.698
LYN_Y	RBFNetwork	0.812	0.850	0.831	0.681
PKB_S	LWL	0.887	0.923	0.905	0.812
PKB_T	Logistic	1.000	1.000	1.000	1.000
SYK_Y	NaiveBayes	0.927	0.964	0.946	0.894
Tec_Y	NaiveBayes	0.906	0.900	0.903	0.815
ZAP70_Y	MultilayerPerceptron	0.967	1.000	0.983	0.969
Other_S	SimpleCart	0.635	0.722	0.678	0.362
Other_T	LADTree	0.502	0.785	0.643	0.300
Other_Y	NaiveBayes	0.714	0.706	0.710	0.421
